# Differential Activation of Programmed Cell Death in Patients with Severe SARS-CoV-2 Infection

**DOI:** 10.21203/rs.3.rs-3059466/v1

**Published:** 2023-06-27

**Authors:** Ashligh Riegler, Paul Benson, Kenneth Long, Sixto Leal

**Affiliations:** The University of Alabama at Birmingham; The University of Alabama at Birmingham; The University of Alabama at Birmingham; The University of Alabama at Birmingham

## Abstract

SARS-CoV-2 (SARS-2) causes severe lower airway disease and death in a subset of patients. Knowledge on the relative contribution of programmed cell death (PCD) to lung pathology is limited to few human autopsy studies with small sample size/scope, in vitro cell culture, and experimental model systems. In this study, we sought to identify, localize, and quantify activation of apoptosis, ferroptosis, pyroptosis, and necroptosis in FFPE lung tissues from patients that died from severe SARS-2 infection (n=28) relative to uninfected controls (n=13). Immunofluorescence (IF) staining, whole-slide imaging, and Image J software was used to localize and quantify expression of SARS-2 nucleoprotein and the following PCD protein markers: cleaved Caspase-3, pMLKL, cleaved Gasdermin D, and CD71, respectively. IF showed differential activation of each PCD pathway in SARS-2 infected lungs and dichotomous staining for SARS-2 nucleoprotein enabling distinction between high (n=9) vs low viral burden (n= 19). No differences were observed in apoptosis and ferroptosis in SARS-2 infected lungs relative to uninfected controls. However, both pyroptosis and necroptosis were significantly increased in SARS-2 infected lungs. Increased pyroptosis was observed in SARS-2 infected lungs, irrespective of viral burden, suggesting an inflammation-driven mechanism. In contrast, necroptosis exhibited a very strong positive correlation with viral burden (R2=0.9925), suggesting a direct SARS-2 mediated effect. These data indicate a possible novel mechanism for viral-mediated necroptosis and a potential role for both lytic programmed cell death pathways, necroptosis and pyroptosis, in mediating infection outcome.

## Introduction

Severe acute respiratory syndrome coronavirus 2 (SARS-2) is an enveloped positive-sense RNA betacoronavirus causing a wide spectrum of disease severity, culminating in pneumonia, Acute Respiratory Distress Syndrome (ARDS), and death ^1, 2, 3^. As of March 2023, SARS-2 has infected over seven hundred million individuals and COVID-19, the disease attributed to SARS-2 infection, has resulted in more than six million deaths (see link for continuous updates https://covid19.who.int). Despite the current availability of vaccines with 70–95% efficacy, through the combined effect of low vaccination rates (~60%; even lower for boosters), waning immunity, and the continued emergence of novel immune-evading variants, SARS-2 is now endemic in the US and epidemic in other countries, notably China ^4^. Individuals of all ages and health status are susceptible, however, unvaccinated individuals, as well as persons with advanced age, obesity, diabetes, sickle cell disease, multiple comorbidities, critical illness, and immunosuppression are at highest risk of progression to severe disease ^5^.

SARS-2 mediated ARDS is a life-threatening pulmonary condition characterized by excessive lung inflammation, tissue damage, airway edema, hemorrhage, and profound hypoxia with high mortality (40–60%) ^6^. Pulmonary epithelial cell death contributes to high mortality but the relative contribution of specific forms of programmed cell death during SARS-2 infection is not well defined. Previous studies on human tissues exhibited low sample size and findings in experimental *ex-vivo*, *in-vitro*, and animal infection models do not fully recapitulate the lungs of patients with severe SARS-2 infection ^7^. Experimental *in vitro* cell culture studies have shown that SARS-2 infection induces apoptosis in bronchial epithelia, microvascular endothelial cells, platelets, macrophages, dendritic cells, and T cells ^7, 8, 9, 10, 11, 12^, necroptosis in lung epithelial cells, platelets, and neutrophils ^8, 13, 14^, as well as pyroptosis in lung epithelial cells ^15^. Infected animal models have shown that SARS-2 infection induces necroptosis in the lungs of infected mice ^10^, ferroptosis in the lungs of infected hamsters ^16, 17^, and apoptosis in the lungs of non-human primates ^18^. Examination of blood and serum from SARS-2 infected patients has also shown increased expression of the necroptosis signaling protein, RIPK3, as well as pyroptosis-associated inflammatory markers (IL-1β, IL-18, and M65-antigen) and indicators of ferroptosis (GPX4, FTH1, FTL, and SAT1)^15, 19, 20^. Finally, limited human autopsy studies of infected patients with small sample size/scope have shown activation of ferroptosis in cardiac tissue ^21^, pyroptosis in pulmonary macrophages ^22^, as well as necroptosis signaling (pRIPK1, pRIPK3, and pMLKL) by western blot analysis and histology in BAL protein extracts, serum, platelets, and lung epithelial cells ^8, 10, 13, 14, 19, 23^.

PCD can be broadly categorized as non-lytic, including apoptosis and autophagy, and lytic, including all forms of programmed necrosis, such as necroptosis, pyroptosis, and ferroptosis ^24^. Non-lytic PCD, including apoptosis and autophagy, occurs when affected cells re-package their intracellular contents prior to release, reducing the overall inflammatory response. Both apoptosis and autophagy are mediated primarily by intracellular caspases, with apoptosis executed by the cleavage of caspase 3 and autophagy executed by various phagosomal and lysosomal maturation signals. Apoptosis results in degradation of chromosomal DNA as well as nuclear and cytoskeletal proteins, cellular re-packaging and cytomorphologic changes, culminating in the formation of apoptotic vesicles ^25^. Although non-lytic PCD results in minimal inflammation, dependent on the extent of involvement, it can mediate significant effects on lung pathology (e.g. endothelial cell apoptosis resulting in alveolar hemorrhage) ^18^. In contrast, lytic PCD including all forms of programmed necrosis, like ferroptosis, pyroptosis, and necroptosis, results in significant inflammation as result of the release of damage-associated molecular patterns (DAMPs), following cellular dissolution, that promote leukocyte recruitment and activation ^24^. Ferroptosis is a form of lytic PCD, which results from the accumulation of oxidized lipids, often as a result of ferritin breakdown, releasing ferric iron (Fe^3+^) into the cytosol where it reacts with available reactive oxygen species through the Fenton reaction, ultimately resulting in the formation of oxidized lipids. While there is no identified specific molecular executioner of ferroptosis, it is characterized by upregulation of the transferrin receptor (CD71) and iron-dependent lipid peroxidation, which promotes membrane rigidity and damage, culminating in cellular lysis ^26^. Unlike ferroptosis, pyroptosis is carried out by a cascade of cellular caspases, primarily caspase 1, and the formation of the cellular “inflammasome” (pro-caspase-1, ASC, and NLRP3), ultimately orchestrating the assembly of lethal pores constructed of activated/cleaved gasdermin D (cGasdD) on the cell membrane ^27^. Similarly, necroptosis is carried out by the formation of large membrane pores. Necroptosis relies on the activation (by phosphorylation) of the executioner protein MLKL (mixed lineage kinase domain-like protein) by the receptor-interacting serine-threonine kinase 3 (RIPK3). Phosphorylated MLKL (pMLKL) forms pores on the cellular surface, resulting in membrane dissolution and cellular necrosis ^28^. To add to this complexity, a continuum of PCD, termed PANoptosis, has also been described and *in vitro* studies indicate that SARS-2 ORF3a is able to intercalate into host cell membranes and amplify host lytic PCD ^23, 29, 30^.

Utilizing unique access to a large archive of post-mortem lung tissue, the current study sought to identify, localize, and quantify activation of apoptosis, ferroptosis, pyroptosis, and necroptosis in FFPE lung tissues from patients that died from severe SARS-2 infection (n=28) relative to uninfected controls (n=13). These data provide significant evidence in infected human samples of a potential role for both necroptosis and pyroptosis in mediating lung pathology during severe SARS-2 infection. Additionally, we show evidence that pyroptosis in the severe COVID lung is inflammation driven, whereas, necroptosis is a viral driven phenomenon. These insights in human tissues suggest the existence of a novel mechanism in which SARS-2 directly mediates or enhances necroptosis in infected host cells.

## MATERIALS/SUBJECTS AND METHODS

### Ethical Approval

This study was approved by the UAB Institutional Review Board (IRB # 300008563).

### Tissue Processing

Formalin fixed paraffin-embedded (FFPE) lung tissue from patients that died from severe SARS-2 infection (n=28) between January 2020 and January 2022 and control tissues (n=13) were included. Autopsies were performed via standard of care procedures in the UAB Department of Pathology. Briefly, lungs were inflated with 10% formalin in phosphate buffered saline and fixed for 24 hours prior to processing and sectioning. Five μm thick sections were cut for special stains including hematoxylin and eosin for evaluation of lung pathology by bright-field microscopy and immunofluorescence (IF) staining as described below.

### Immunofluorescence Staining

Tissue sections were baked at 60°C for 30 minutes to loosen paraffin from tissues followed by cooling at room temperature. Slides were then de-paraffinized and rehydrated with the following sequence of immersions: 10 min in 100% Xylene Substitute, 5 min in 1:1 Xylene Substitute and 100% Ethanol, 5 min in 95% Ethanol, 5 min in 75% ethanol, 5 min in 50% Ethanol, and 10 min in phosphate-buffered saline (PBS). Slides were then placed in TRILOGY^™^ (Cell Marque, Rocklin, CA) and steamed for 20 min for antigen retrieval. After cooling, slides were rinsed for 5 min in PBS, permeablized with 0.05% Triton X-100 in PBS for 5 min, and rinsed again in PBS. Tissue sections were blocked using 3% BSA in PBS for 40 min at room temperature prior to the addition of primary antibody (see **Supplementary table 1** for antibody list and dilutions). For ferroptosis, it is important to note that a specific antibody clone (3F3-FMA) targeting the transferrin receptor (CD71) was used (X). Following primary antibody staining and three washes with PBS, secondary antibody stain solution was added. Tissue slides were then washed again with PBS and nuclei counterstained using the NucBlue^™^ Fixed Cell ReadyProbes^™^ Reagent (Life Sciences) per manufacturer’s instructions. Tissues were cover slipped using FluorSave^™^ mounting media (Millipore Sigma, Burlington, MA) and stored at 4°C in the dark prior to imaging within 48h.

### Full Slide Imaging

Stained slides were imaged using a Leica LMD6 microscope with LASX software. Tiled images were obtained at 100X magnification with fixed exposure, gain, and signal threshold settings for each target. Images were then stitched together and exported as a TIFF for further analysis.

### Image Analysis and Statistics.

ImageJ 1.53k software (National Institutes of Health) was used to determine mean and integrated signal intensity. Signal co-localization was determined using the Image J Co-localization plugin (Pierre Bourdoncle, Institut Jaques Monod, Service Imagerie, Paris). Statistical comparisons were carried out in GraphPad Prism 9 (GraphPad Software, San Diego, CA). For comparisons between groups, the two-tailed Student’s t test was used for normally distributed data. Pearson or Spearman’s correlation were used to identify the association between ranked variables, as indicated. For clinical variables, ANOVA with Fisher’s Least Significant Difference (LSD) test was used to determine statistical significance in continuous variables and the Chi Square test used for qualitative variables. Statistical tests are denoted in the associated figure legends. Data represent mean ± standard error of the mean (SEM) unless otherwise noted. A p value ≤ 0.05 was considered significant.

## RESULTS

### Lungs from patients that died from severe SARS-2 infection exhibit distinct microscopic findings.

Table 1 outlines the characteristics of patients that died from severe SARS-2 infection (n=28) relative to individuals that died from a non-respiratory cause of death (n=13). No statistically significant differences were observed in age, sex, or race. Patients that died from severe SARS-2 infection exhibited a comparable incidence of comorbid conditions to the control cohort, except for a statistically significant increase in the likelihood of a cancer diagnosis in the control vs SARS-2 cohort (46% vs 4%), due to selection bias of available control samples. The SARS-2 cohort consists of both individuals that died within 21 days (n=16; considered acute) and patients that died ≥ 21 days (n=12; chronic) after diagnosis of viral infection. Twenty one days was chosen based on literature consensus of the time required for a healthy host to develop adaptive immunity and cease viral shedding^31, 32, 33^. Consistent with progressive respiratory decline, patients that died from severe SARS-2 infection exhibited a significant increase in ICU admittance/duration, mechanical ventilation/duration, administration of pressors, extracorporeal membrane oxygenation, continuous renal replacement therapy, and treatment with immunosuppressive agents. Additionally, 11/28 (39%) of patients developed 2° bacterial lung infection. Of those, 45.5% with Gram negative, 27.3% with Gram positive, and 27.3% with mixed infections of both Gram negative and positive bacteria. Factors listed on the pathology report as contributing to death include a significant increase in pneumonia in the SARS-2 vs control cohort (100% vs 0%). A total of 25/28 (89%) of individuals in the SARS-2 cohort were unvaccinated, and 3/28 (11%) whose vaccination status was unknown. In patients that died of acute vs chronic SARS-2 infection, 14/16 (88%) vs 11/12 (92%) were unvaccinated, and 2/16 (13%) vs 1/12 (8%) had an unknown status. Due to limited samples from vaccinated patients, we were not able to compare vaccination status to other variables, however these relationships likely exist and contribute to disease presentation. **Supplementary Table S2** shows additional correlations between patient demographics, comorbidities, clinical course/intervention, and factors contributing to death.

Table 2 highlights key microscopic features in the lungs of patients that died from severe SARS-2 infection (n=28) relative to control lung tissues (n=13) from patients with a non-respiratory cause of death. Each case was evaluated by the attending autopsy pathologists as per standard of care and re-reviewed by a blinded infectious disease pathologist to determine the total # of cases with each microscopic finding and quantify the severity of a subset of microscopic findings using a scale from 0 (trace) to 4 (most severe). Consistent with severe lung pathology, the SARS-2 cohort exhibited statistically significant increases in the following microscopic features: diffuse alveolar damage, emphysema, inflammation, hemorrhage, type II pneumocyte hyperplasia, hyaline membranes, and alveolar fibroblast proliferation. The # of cases with microthrombi and edema were also increased in SARS-2 vs control infection cohorts but did not reach statistical significance. Notably, 18/28 (64%) of SARS-2 + cases exhibited macrophage-predominant inflammation, 4/28 (14%) showed neutrophil predominance, and 6/28 (21%) exhibited both. Of the 11 patients noted to have 2° bacterial infection 7/11 (64%) showed macrophage predominance, 1/11 (9%) showed neutrophil predominance, and 3/11 (27%) showed both. Likewise, of patients that died of acute vs chronic SARS-2 infection, 11/16 (69%) vs 7/12 (58%) exhibited macrophage predominance, 1/16 (6%) vs 3/12 (25%) showed neutrophil predominance, and 2/16 (13%) vs 1/12 (8%) showed both. Statistically significant increases in severity scores were noted in the SARS-2 cohort for the following parameters: inflammation, hemorrhage, type II pneumocyte hyperplasia, and hyaline membranes. A trend towards a statistically significant increase was also observed for emphysema, edema, and interstitial fibrosis. **Supplementary Table S2** shows additional correlations between patient characteristics and microscopic findings.

### Patients that died from severe SARS-2 infection exhibit significant lung pathology with variable viral burden.

**Figure 1.**
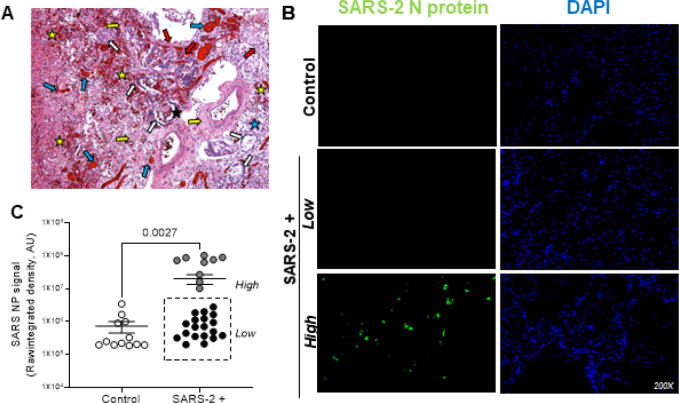
Patients that died from severe SARS-2 infection exhibit significant lung pathology with variable viral burden. **A.** Representative H&E image (100X) from a patient that died from severe SARS-2 infection. Diffuse alveolar damage is noted throughout. The white arrows indicate type II pneumocyte hyperplasia, yellow arrows indicate loose interstitial fibrosis, blue arrows indicate vascular congestion, black starshighlight intra-alveolar fibrinous exudates, and yellow stars indicate hemorrhage. **B.** Representative fluorescent images show variable staining with antibodies targeting SARS-2 nucleoprotein (green) in SARS-2-positive lungs and no signal in control lung tissues (200X; DAPI counterstain). **C.** Image J was used to quantify raw integrated intensity in control (open circles) versus SARS-2+ (enclosed circles) lung tissues. The dashed box highlights a dichotomous population of patients with severe COVID, exhibiting either high or low SARS-2 burden in the lungs at the time of death. T-tests were used to determine statistical significance set at a p-value ≤0.05.

Pathological features of severe SARS-2 infection have previously been described in post-mortem autopsy studies ^34^. Figure 1A shows a representative image of an H&E stained lung section from a patient that died of severe SARS-2 infection. Prominent microscopic features include diffuse alveolar damage, type II pneumocyte hyperplasia, alveolar fibroblast proliferation, vascular congestion, intra-alveolar fibrinous exudates, hemorrhage, and inflammation. Table 2 denotes the total # of cases per cohort with each microscopic entity and quantifies their relative severity. Given wide variability in the total # and severity of microscopic features between patients that died of severe SARS-2 infection, we sought to localize and quantify viral burden in the lungs utilizing immunofluorescence staining. Figure 1B shows representative fluorescent images of SARS-2 nucleoprotein (green) in SARS-2-positive lungs and no signal in control lung tissues. Image overlays with DAPI-stained slides (not shown) localize SARS-2 nucleoprotein to pulmonary epithelial cells that line both alveoli, bronchioles, and larger airways. To determine viral lung burden, SARS-2 nucleoprotein staining was quantified utilizing Image J software. Figure 1C highlights a dichotomous population of patients that died with severe SARS-2 infection harboring either high (n=9) or low (n=19) SARS-2 nucleoprotein/viral load.

**Supplementary Table S2 and S3** highlight correlations between patient demographics, comorbidities, clinical course/intervention, factors contributing to death, and microscopic findings for SARS-2+ patients with high versus low viral burden. **Table S3** denotes statistically significant increases in diabetes, cardiovascular disease, and a history of smoking, chronic obstructive pulmonary disease, or asthma in patients with high vs low SARS-2 burden at the time of death. **Table S2** showsa moderate to strong Spearman’s rank correlation between the factors outlined above and SARS-2 nucleoprotein. **Table S4** highlights key microscopic features in the lungs of patients that died from severe SARS-2 infection with high versus low viral burden. Statistically significant differences include an increased % of cases with documented microthrombi in patients with high SARS-2 burden (33% vs 5%) and decreased type II pneumocyte hyperplasia in this same cohort (44% vs 95%). No statistically significant differences were observed in the severity of microscopic features, however, **Table S2** shows a moderate to strong Spearman’s correlation between diffuse alveolar damage (r:0.39), airway inflammation (r:0.45), and SARS-2 nucleoprotein. **Table S2** also shows a high correlation of SARS-2 nucleoprotein with chronic infection >/= 21 days (r:0.53).

### Apoptosis and ferroptosis are detected but not significantly upregulated in the lungs of patients that died from severe SARS-2 infection relative to control lung tissue.

**Figure 2.**
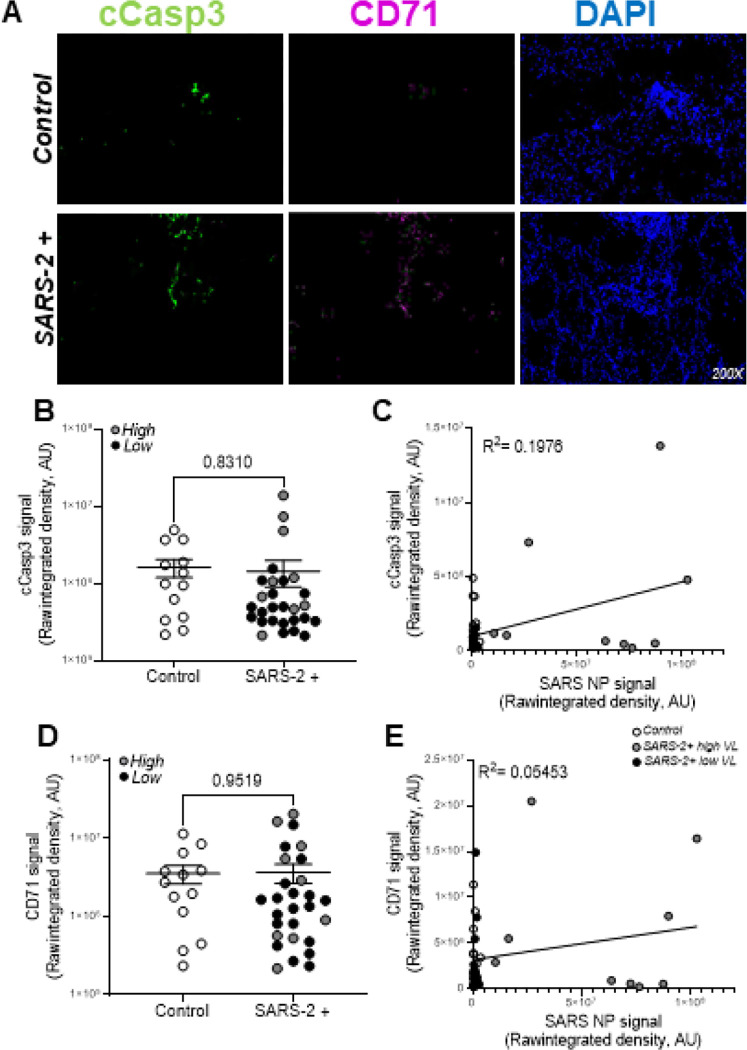
Apoptosis and ferroptosis are detected but not significantly upregulated in the lungs of patients that died from severe SARS-2 infection relative to control lung tissue. **A.** Representative fluorescent images show comparable cleaved caspase 3 (apoptosis marker) and CD71 (ferroptosis marker) in the lungs of patients that died from severe SARS-2 infection relative to control lung tissues. **B.** Image J was used to quantify raw integrated intensity for cleaved caspase 3 in control (open circles) versus SARS-2+ (enclosed circles) lung tissues. **C.** Pearson correlation and linear regression showed no correlation between cleaved caspase 3 and SARS-2 burden. Similarly, **D.** Image J was used to quantify CD71 raw integrated intensity and **E.** linear regression showed no correlation between CD71 and viral burden. T-tests or one-way ANOVA with Tukey’s post-test were used to determine statistical significance set at a p-value ≤ 0.05.

To determine the relative contribution of programmed cell death to lung pathology we utilized immunofluorescence staining, whole-slide imaging, and Image J software to localize and quantify expression of PCD protein markers in FFPE lung tissues from patients that died from severe SARS-2 infection (n=28) relative to uninfected controls (n=13). Figure 2A shows representative fluorescent images of cleaved caspase 3 (apoptosis marker; green) and CD71 (ferroptosis marker; purple) in the lungs of patients that died from severe SARS-2 infection relative to control lung tissues. Image overlays with DAPI-stained slides (not shown) localize both PCD markers to pulmonary epithelial cells. Figure 2B shows no statistically significant difference in cleaved caspase 3 signal in control (open circles) versus SARS-2+ (enclosed circles) lung tissues. Grey and black circles represent patients with high and low SARS-2 lung burden, respectively. No difference in cleaved caspase 3 signal was observed in samples with high vs low SARS-2 burden. In Figure 2C, Pearson correlation and linear regression analyses show no correlation between cleaved caspase 3 and SARS-2 nucleoprotein/viral load. Similarly, Figure 2D shows no statistically significant difference in CD71 signal in control (open circles) versus SARS-2+ (enclosed circles) lung tissues and Figure 2E shows no correlation between CD71 and SARS-2 burden. **Table S2** shows an unexpected strong Spearman’s rank correlation between CD71 (r:0.81) and cleaved caspase 3.

### Pyroptosis is significantly upregulated in the lungs during severe SARS-2 infection but does not correlate with viral burden

**Figure 3.**
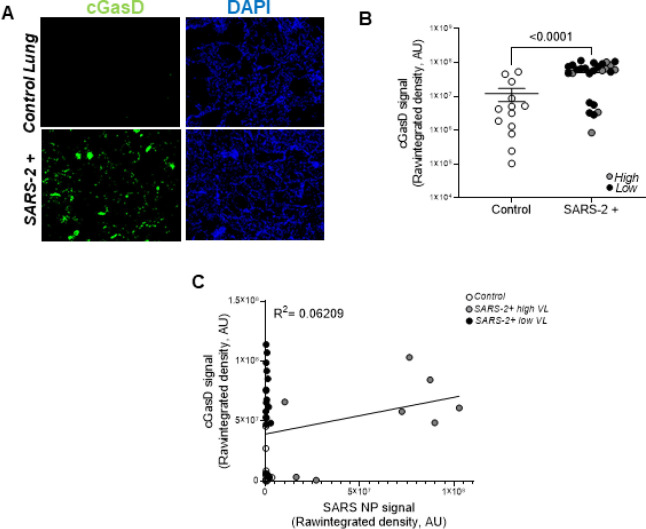
Pyroptosis is significantly upregulated in the lungs during severe SARS-2 infection but does not correlate with viral burden. **A.** Representative fluorescent images show increased cleaved gasdermin D (pyroptosis marker) in the lungs of patients that died from severe SARS-2 infection relative to control lung tissues. **B.** Quantification of raw integrated intensity for cleaved gasdermin D shows significantly increased signal in SARS-2 infected lungs relative to control lung tissues. **C.** Pearson correlation and linear regression show no correlation between cleaved gasdermin D and SARS-2 burden. T-tests or one-way ANOVA with Tukey’s post-test analyses were used to determine statistical significance set at a p-value ≤ 0.05.

Figure 3A shows representative fluorescent images of cleaved gasdermin D (pyroptosis marker; green) in the lungs of patients that died from severe SARS-2 infection relative to control lung tissues. Cleaved gasdermin D signal is prominent in SARS-2 infected lungs relative to controls and the majority of it localizes to pulmonary epithelial cells (not shown). Figure 3B shows a statistically significant increase in cleaved gasdermin D signal in SARS-2+ (enclosed circles) vs control (open circles) lung tissues. Grey and black circles, representing patients with high and low SARS-2 lung burden, are evenly distributed and Figure 3C demonstrates no correlation between cleaved gasdermin D and SARS-2 nucleoprotein/viral load. Increased gasdermin D signal that does not correlate with SARS-2 burden suggests a viral-independent mechanism, possibly inflammation, as the major driver of pyroptosis in patients with severe COVID. **Table S2** shows a strong Spearman’s rank correlation for ICU admittance (r:0.55), airway inflammation (r:0.45), macrophage predominant inflammation (r:0.49), hemorrhage (r:0.41), type II pneumocyte hyperplasia (r:0.45), and hyaline membranes (r:0.58) with cleaved gasdermin D, suggesting a significant role in clinical disease severity, lung pathology, and infection outcome.

### Necroptosis is significantly upregulated in the lungs during severe SARS-2 infection and exhibits a strong positive correlation with viral burden.

**Figure 4:**
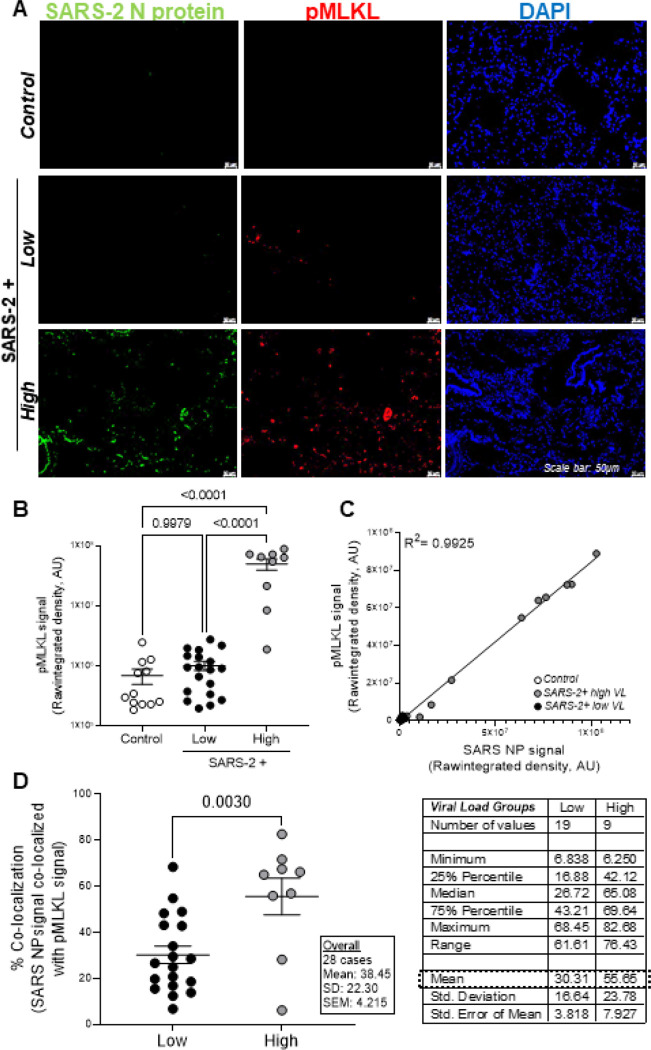
Necroptosis is significantly upregulated in the lungs during severe SARS-2 infection and exhibits a strong positive correlation with viral burden. **A.** Representative fluorescent images show phosphorylated MLKL (necroptosis marker) in the lungs of patients that died from severe SARS-2 infection with minimal detection in control lung tissues. Additionally, increased signal was observed in SARS-2+ lung tissues with high viral burden relative to low viral burden. **B.** Quantification of raw integrated intensity for pMLKL shows significantly increased signal in SARS- 2-infected lungs with high viral burden relative to samples with low viral burden and control lung tissues. **C.** Pearson correlation and linear regression show a strong positive correlation between pMLKL and SARS-2 burden. **(D)** Image J was used to quantify % co-localization of pMLKL with SARS-2 nucleoprotein. T-tests or one-way ANOVA with Tukey’s post-test were used to determine statistical significance set at a p-value ≤ 0.05.

Figure 4A shows representative fluorescent images of SARS-2 nucleoprotein (green) and phosphorylated MLKL (necroptosis marker; red) in the lungs of patients that died from severe SARS-2 infection relative to control lung tissues. Increased pMLKL signal was observed in SARS-2+ lung tissues with high viral burden relative to tissues with low viral burden and the majority of it localized to pulmonary epithelial cells (not shown). Figure 4B shows a statistically significant increase in pMLKL in SARS-2-infected lungs with high viral burden relative to samples with low viral burden and control lung tissues. In Figure 4C, Pearson correlation and linear regression analyses were used to show a strong positive correlation (R^2^ = 0.9925) between pMLKL and SARS-2 burden. **Table S2** shows a strong Spearman’s rank correlation between pMLKL and SARS-2 nucleoprotein (r:0.92). To assess if cells undergoing necroptosis are the same as those infected with SARS-2, we determined the percent signal co-localization of both targets using ImageJ software. Figure 4D shows a statistically significant increase in the percent co-localization of pMLKL with SARS-2 nucleoprotein in patients with high versus low viral burden. Interestingly, a strong correlation was also identified between pMLKL and chronic infection, >/= 21 days (r:0.60), suggesting that a high viral load over a long period of time, may be the major driver of necroptosis in patients that died from severe SARS-2 infection. The strong positive correlation and co-localization between pMLKL and SARS-2 burden, suggests that, unlike pyroptosis, a direct viral-mediated mechanism drives necroptosis in patients with severe COVID.

### Patients with SARS-2 infection and 2° bacterial pneumonia exhibit no change in the lung programmed cell death profile relative to SARS-2 only infection.

Table 1 shows that 11/28 (39%) of patients that died from severe SARS-2 infection had evidence of 2° bacterial lung infection during their hospitalization. To determine if 2° infection impacts the programmed cell death profile in patients that died from severe SARS-2 infection, we split the SARS-2 cohort into patients with (n= 11) or without (n=17) evidence of 2° bacterial lung infection and reanalyzed the data for each independent variable described above. Consistent with SARS-2 as the major mediator of the lung microenvironment in this patient population, **Figure S1** shows no statistically significant difference between patients with SARS-2-only versus 2° infection for the following variables: **Figure S1A** SARS-2 nucleoprotein (N protein), **Figure S1B**. cleaved caspase-3 (cCasp3), **Figure S1C** transferrin receptor (CD71), **Figure S1D** cleaved gasdermin D (cGasD), and **Figure S1E** phosphorylated MLKL (pMLKL). Although not statistically significant, **Figure S1C** shows a trend towards decreased CD71 signal during 2° infection, which we hypothesize may be mediated by bacterial consumption of excess iron in virus-damaged lungs. **Table S2** shows additional correlations between patient characteristics and microscopic findings.

## DISCUSSION

SARS-2 mediates significant tissue damage and inflammation during lung infection ^34, 35^. In this study, we sought to identify, localize, and quantify activation of apoptosis, ferroptosis, pyroptosis, and necroptosis in FFPE lung tissues from patients that died from severe SARS-2 infection (n=28) relative to uninfected controls (n=13). To our knowledge, this is the first and largest evaluation of multiple programmed cell death pathways during severe COVID using patient tissues ^10, 18, 23^. Immunofluorescence (IF) staining showed differential activation of each PCD pathway in SARS-2 infected lungs and dichotomous staining for SARS-2 nucleoprotein enabling distinction between lungs with high (n=9) vs low viral burden (n= 19). No differences were observed in apoptosis and ferroptosis in SARS-2 infected lungs relative to uninfected controls. However, both pyroptosis and necroptosis were significantly increased in SARS-2 infected lungs. Increased pyroptosis was observed in SARS-2 infected lungs, irrespective of viral burden, suggesting an inflammation-driven mechanism. In contrast, necroptosis exhibited a very strong positive correlation with viral burden (R^2^=0.9925), suggesting a possible novel direct mechanism for viral-mediated necroptosis.

Consistent with progressive respiratory decline, patients that died from severe SARS-2 infection exhibited a significant increase in ICU admittance/duration, mechanical ventilation/duration, pressors, ECMO, and CRRT. Similarly, the SARS-2 cohort also exhibited significant increases in diffuse alveolar damage, emphysema, inflammation, hemorrhage, type II pneumocyte hyperplasia, hyaline membranes, and alveolar fibroblast proliferation, accordant with severe lung pathology. These microscopic findings are consistent with the literature showing extensive impairment of alveolar epithelial cells, hyaline membrane formation, focal hemorrhage, diffuse alveolar damage, intra-alveolar inflammation, and hyperplasia of type II pneumocytes ^36, 37^. Interestingly, previous studies investigating the inflammatory response to SARS-2 infection differ in their identifications of macrophage or neutrophil predominance, with some indicating the systemic predominance of neutrophils or neutrophil-related markers, like myeloperoxidase and cell-free DNA associated with citrullinated histones, as an indicator of disease severity or enhanced risk for vascular manifestations^37, 38^. Others have identified connections between a systemic dominance of macrophages or macrophage-associated cytokine signals, such as IL-6 and CCL-2, and severe SARS-2 disease manifestations, including neurological symptoms ^39, 40^. Of the 28 SARS-2+ cases examined in this study, 64% (18/28) exhibited macrophage-predominant inflammation, whereas only 4/28 (14%) showed neutrophil predominance, and 6/28 (21%) exhibited airway exudates with both macrophages and neutrophils. Macrophage predominance relative to neutrophils may ultimately increase susceptibility to 2° fungal and bacterial infections, the latter of which we observed in 11/28 of the cases assessed in our cohort ^41^. Notably, of patients with 2° bacterial infection, 7/11 (64%) showed macrophage predominant inflammation, only 1/11 (9%) had neutrophil predominant inflammation, with 3/11 (27%) exhibiting both.

Microscopically, a statistically significant increase was observed in the % of cases with documented microthrombi in patients with high SARS-2 burden (33% vs 5%) and decreased type II pneumocyte hyperplasia in this same cohort (44% vs 95%) as well as a moderate to strong Spearman’s correlation between diffuse alveolar damage (r:0.39) and airway inflammation (r:0.45) with SARS-2 nucleoprotein. These microscopic findings are indicators of disease severity and unsurprisingly, previous work has shown that viral load is associated with disease severity and risk of mortality ^42^.

Given the total # and wide variability in the severity of microscopic features between patients that died of severe SARS-2 infection, we sought to localize and quantify viral burden in the lungs utilizing immunofluorescence staining and identified a dichotomous population of patients that died with severe SARS-2 infection harboring either high or low viral load. This variability in viral lung burden is consistent with prior studies utilizing quantitative PCR and in situ RNA hybridization ^43, 44, 45, 46^ Viral burden also correlated with ICU duration (r: 0.33) as well as the total duration of infection (>/= 21 days; r: 0.53). This may be due to delayed viral clearance as a result of intrinsic or iatrogenic immune impairment due to corticosteroid therapy, which is known to prolong viral infection and render patients susceptible to 2° infection ^47, 48^. Consistent with the literature, we also show a statistically significant increase in diabetes, cardiovascular disease, and a history of smoking, chronic obstructive pulmonary disease, or asthma in patients with high vs low SARS-2 burden at the time of death ^49, 50, 51^. The majority (25/28; 89%) of patients in the SARS-2 cohort, as well all of the patients with high viral burden (9/9; 100%), were unvaccinated. Despite the current availability of vaccines with high efficacy, the low vaccination rates, waning immunity, and continued emergence of novel immune-evading variants continue to cause severe infection, particularly, in high risk patients ^4, 52^.

Apoptosis is initiated upon cleavage of intracellular caspase-3 and results in a clean form of cell death, in which cellular contents are neatly packaged and degraded within cells. In this study, we show that although apoptosis is occurring in SARS-2 infected lungs, the amount detected is not significantly increased relative to control tissues. This is consistent with findings by Li *et.al.* (n=1) and Liu *et.al.* (n= 4) in SARS-2 infected FFPE lung tissues ^10, 18^. However, in addition to significantly more cases (n=28), our approach targeting cleaved caspase-3, bypasses the ambiguity of TUNEL staining associated with off-target detection of non-apoptotic DNA fragmentation ^53^. Our findings are also consistent with SARS-2 induced apoptosis in monocytes and T cells isolated from human peripheral blood as well as in bronchial and endothelial cells in experimental human lung organoids, non-human primate infection models, murine and *in vitro* studies ^8, 9, 11, 13, 16, 18^.

Interestingly, **Table S2** shows an unexpected strong positive correlation (r: 0.81) between cleaved caspase-3 and CD71. While there is not a direct link between apoptosis and ferroptosis identified in the literature at this time, both PCD pathways can be initiated by intracellular oxidative stress ^54^. Similarly, both apoptosis and ferroptosis can be inhibited by treatments which regulate intracellular ROS production, mitochondrial dysfunction, and glutathione activity ^55^.

Ferroptosis is characterized by iron-dependent lipid peroxidation, membrane rigidity, and host cell lysis ^56, 57^. Consistent with the literature, we show significant lung damage including cell-lysis, hemorrhage, hemolysis, and inflammation in the lungs of patients with severe SARS-2 infection. Our lab has also shown statistically significant increases in iron in bronchoalveolar lavage samples from patients with severe viral infection relative to non-viral pneumonia (data not shown). Together, high iron and inflammation-mediated oxidative stress during severe SARS-2 lung infection, suggests a potentially major role for ferroptosis in SARS-2 mediated lung pathology. However, unexpectedly, we show that similar to apoptosis, ferroptosis occurs but is not significantly increased in SARS-2 infected lungs with no correlation to viral load. These results suggest that the lung may not have the same ferroptosis response to SARS-2 infection as other tissues, including the heart and kidney ^21, 22, 58, 59, 60, 61, 62, 63^.

Notably, the 3F3-FMA antibody clone targeting the transferrin receptor, CD71, was used as an indicator for ferroptosis in this study. CD71, is upregulated in the setting of high extracellular iron to help sequester and internalize iron-bound transferrin until extracellular iron levels drop. It accumulates in the membranes of cells undergoing ferroptosis, via an unknown mechanism thought to be driven by altered iron metabolism. In a screen of ~4500 antibodies isolated from mice immunized with ferroptotic human cells, this specific antibody clone targeting CD71 was shown to accurately distinguish between ferroptosis and other forms of programmed cell death, including apoptosis ^57^. In this study, this antibody clone did not detect increased CD71 in lung tissue from SARS-2 infected patients. Future studies will incorporate the evaluation of additional ferroptosis and lipid peroxidation markers including malondialdehyde (MDA; 1F83 clone) and 4-hydroxynonenal (4-HNE) ^64, 65^.

Pyroptosis is activated by pro-inflammatory stimuli, which triggers inflammasome and caspase-dependent cleavage of the pore forming effector protein gasdermin D ^66^. In this study, we show that cleaved gasdermin D is prominent in SARS-2 infected lungs relative to controls with no correlation to SARS-2 nucleoprotein/viral load suggesting a viral-independent mechanism, possibly inflammation, as the major driver of pyroptosis. Additionally, pyroptosis correlates strongly with ICU admittance (r: 0.55), airway inflammation (r: 0.45), macrophage predominant inflammation (r: 0.49), hemorrhage (r: 0.41), type II pneumocyte hyperplasia (r: 0.45), and hyaline membranes (r: 0.58). These data are consistent with a previously published small study (n=6) showing increased cGasD in FFPE lung tissues of patients who died from SARS-2 infection as well as elevated IL-1β and IL-18 in BALs, peripheral blood and circulating immune cells during SARS-2 infection ^22, 29, 67, 68, 69, 70, 71, 72, 73^. Consistent with inflammation-driven pyroptosis, *in vitro* studies highlight autocrine/paracrine cytokine signaling as the major mediator of NLRP3 activation and pyroptosis during SARS-2 infection ^67, 74^. Our finding that pyroptosis is associated with increased clinical and microscopic disease severity is consistent with the reported predictive value of systemic cleaved caspase-1 and IL-18 in patients for progression to severe or fatal disease ^29^.

Necroptosis occurs in cells unable to undergo apoptosis and is mediated by the phosphorylation of RIPK1, RIPK3, and the pore-forming effector protein MLKL^28^. In this study, we show a statistically significant increase in pMLKL in SARS-2-infected lungs with high vs low viral burden as well as signal co-localization and a strong positive correlation (R^2^ = 0.9925) between pMLKL and SARS-2 consistent with direct viral-mediated activation of necroptosis. Additionally, we show a strong correlation between pMLKL and chronic infection suggesting viral exposure over a long period of time, drives necroptosis in patients with severe SARS-2 infection. These data are in agreement with a recent publication by *Schifanella et. al.* identifying necroptosis in type II alveolar epithelial cells in FFPE lung samples from patients (n=4) that died from severe SARS-2 infection ^75^. It is also in agreement with upregulated systemic phosphorylated RIPK3 in mechanically-ventilated versus non-ventilated patients ^19, 76^. Viral-driven necroptosis is further supported by numerous publications indicating its evolution as a mechanism to eliminate viral-infected cells unable to undergo apoptosis ^77, 78^. Although studies with the original SARS-1 ORF3a protein show that it can also intercalate into host cell membranes increasing cell permeability and ion perturbations that can amplify host lytic PCD, we did not evaluate this alternative cell death pathway in the current study ^79, 80, 81^.

Our finding of significantly increased activation of both pyroptosis and necroptosis during severe SARS-2 infection suggests the possibility of PANoptosis, a recently identified PCD continuum characterized by TNFα and IFNγ-driven activation of apoptosis, pyroptosis and necroptosis in the same cell ^82^. However, apoptosis was not significantly increased in patients with SARS-2 infection and only a weak correlation (r: 0.14) was identified between pyroptosis and necroptosis, suggesting no role for PANoptosis in our patient cohort. Previous reports have identified PANoptosis in cells following infection by several viruses including Influenza, however, data for its activation during SARS-2 infection is limited ^23, 83, 84, 85, 86, 87^. Although *Schifanella et. al.* detected all 3 PCD pathways in a small number of FFPE tissues (n=4) from SARS-2-infected patients, colocalization analyses were not performed and it is not clear if the same cell was simultaneously undergoing multiple forms of PCD ^75^. Likewise, although *Karki et. al.* report PANoptosis as critical to the formation of SARS-2 mediated cytokine storm, the majority of the study utilizes a murine model of intraperitoneal TNFα and IFNγ injection and an *in vitro* cell line ^23^. Experiments with SARS are limited and only show enhanced survival of infected mice upon neutralization of TNFα and IFNγ ^23, 88, 89^.

SARS-2 mediated cell lysis provides dead cellular material and releases intracellular nutrients that are now available to promote 2° infection. In this study, we show that 11/28 (39%) of patients developed 2° bacterial lung infection including 45.5% with Gram negative, 27.3% with Gram positive, and 27.3% with mixed infections during their hospitalization. The rate of bacterial co-infection in our cohort is consistent with prior autopsy studies ^90, 91^. Of note, despite a reported 5–10% incidence of COVID-Associated Pulmonary Aspergillosis (CAPA) in patients with severe SARS-2 infection, no patients included in the current study developed a 2° mold infection ^92, 93^. At the time of autopsy, all patients with 2° bacterial infection were treated with appropriate antibacterial agents. Although bacteria can independently promote lytic PCD, microscopic evaluation of lungs from patients in this cohort did not identify the characteristic neutrophil predominance expected during bacterial pneumonia, suggesting successful clearance prior to sampling. Additionally, no difference was observed in lung PCD profiles for patients with 2° infection vs SARS-2 only infection. These data suggests that, if bacterial-driven cell death is present, its contribution to the total PCD profile may be limited on the background of a pre-existing and overwhelming SARS-2-damaged lung microenvironment. Alternatively, its detection may require the evaluation of more samples to reach sufficient statistical power.

Despite corticosteroid therapy in the majority (26/28; 93%) of cases, both pyroptosis and necroptosis predominated in the lungs of patients that died from severe SARS-2 infection. This indicates a potential role for adjunct use of pyroptosis and necroptosis inhibitors to block host cell lysis and progression of SARS-2 mediated lung damage. Consistent with a potential therapeutic role for targeting pyroptosis, several small studies utilizing non-specific NALP3 inflammasome inhibitors have shown anti-inflammatory efficacy in patients with SARS-2 infection ^94, 95, 96^. Phase 1 clinical trials with a RIPK3 inhibitor, SAR443122, in patients with COVID are ongoing but *in vitro* studies indicate that necroptosis inhibition promotes the viability of SARS-2 infected host cells. ^97^.

In summary, by evaluating a unique and large cohort of FFPE lung samples, we have identified significant upregulation of both pyroptosis and necroptosis in patients that died from severe SARS-2 infection. This work highlights viral-mediated inflammation as the main driver of pyroptosis, whereas, prolonged SARS-2 infection drives the activation of necroptosis. Both pathways are identified as critical targets whose pharmacologic inhibition may prove significantly helpful in preserving viable lung tissue, blocking 2° infection, and optimizing clinical outcomes in patients infected with SARS-CoV-2.

## Figures and Tables

**Table 1:** Characteristics of patients that died from severe SARS-2 infection and controls

	SARS-2 + (n=28)		Control (n=13)		*p-value*	

**Demographics**						
Age (years)	54.9	-	62.9	-		*0.14*
Male	17	61%	11	85%		*0.16*
Female	10	36%	2	15%		
Caucasian	9	32%	5	38%		*0.69*
Black	17	61%	8	62%		*0.96*
Hispanic/Latinx	2	7%	0	0%		*0.32*
**Co-morbidities**						
BMI	36.3	-	31.9	-		*0.42*
Obesity	20	71%	8	62%		*0.53*
Diabetes	6	21%	6	46%		*0.11*
Hypertension	14	50%	4	31%		*0.25*
Cardiovascular disease	19	68%	5	38%	~	*0.08*
Chronic Kidney Disease	7	25%	1	8%		*0.19*
Smoker/COPD/Asthma	2	7%	2	15%		*0.41*
Cancer	1	4%	6	46%	***	***<0.001***
Immune suppression prior to SARS-2	1	4%	3	23%	~	*0.09*
**Clinical Course/ Intervention**						
SARS-2 infection < 21 days	16	57%	-	-	-	
SARS-2 infection >/= 21 days	12	43%	-	-	-	
ICU admittance	27	96%	4	31%	****	***<0.0001***
ICU duration (days)	21.7	-	1.8	-	~	***0.06***
Mechanical Ventilation (MV)	24	86%	7	54%	*	***0.027***
MV duration (days)	19.0	-	2.4	-	**	***0.002***
Pressors	23	82%	3	23%	***	*0.0003*
ECMO	6	21%	3	23%		*0.91*
CRRT/ dialysis	14	50%	3	23%		*0.10*
Immunosuppressive treatment	26	93%	2	15%	****	***<0.0001***
2° bacterial lung infection’	11	39%	0	0%	**	***0.008***
**Factors Contributing to Death**					-	
Pneumonia	28	100%	*0*	*0%*	****	***<0.0001***
Cardiovascular death	10	36%	8	62%		*0.12*
Multi-organ failure	1	4%	0	0%		*0.49*
Cancer	0	0%	1	8%		*0.14*
Other'	7	25%	5	38%		*0.38*
**Vaccination Status**						
Unvaccinated^^^	25	89%	-	-	-	
Unknown Status	3	11%	-	-	-	

-ECMO- extracorporeal membrane oxygenation; CRRT- continuous renal replacement therapy

#ANOVA with Fisher’s Least Significant Difference (LSD) test was used for continuous variables;Chi Square test was used for qualitative variables

’2° bacterial lung infection include 45.5% Gram negative bacteria (P.aeruginosa, S. maltophilia, K. pneumoniae, K. aerugenes, P. agglomerans, Achromobacter, Acinetobacter, and Enterobacter, ), 27.3% Gram positive pathogens (methicillin-sensitive or methicillin-resistant S. aureus), and 27.3% mixed infection with both Gram negative and Gram positive bacteria (MRSA with K. pneumoniae, MRSA with P. agglomerans, and S. aureus with E. cloaceae).

' Other factors contributing to death include: terminal lung disease, blunt force trauma, renal failure, complications of therapeutic intervention for hepatic cyst, cirrhosis, and sepsis

^ Individuals that died prior to the availability of SARS-2 vaccines were considered unvaccinated

**Table 2:** Microscopic findings in patients that died from severe SARS-2 infection and controls

	SARS-2 + (n=28)			Control (n=13)			*p-value* *(Scores)*		*p-value* *(# cases)*	
	Cases	%	Score^	Cases	%	Score^				
Diffuse alveolar damage	19	68%	-	0	0%	-	-		**	***0.0013***
Emphysematous Changes	15	54%	1.46	2	15%	0.38	~	*0.07*	*	***0.02***
Airway inflammation	28	100%	2.91	3	23%	0.38	****	***<0.0001***	****	***<0.0001***
* Macrophage- predominant^*	18	64%	-	3	100%	-	*-*			*021*
* Neutrophil-* *predominant^*	4	14%	-	0	0%	-	-			*0.48*
* Both^*	6	21%	-	0	0%	-	-			*0.37*
Microthrombi	4	14%	-	0	0%	-	-			*0.15*
Hemorrhage	18	64%	2.00	1	8%	0.23	**	***0.0035***	***	***0.0007***
Edema	15	54%	1.63	3	23%	0.46	~	*0.054*	*~*	*0.07*
Type II pneumocyte hyperplasia	22	79%	3.23	1	8%	0.35	****	***<0.0001***	****	***<0.0001***
Hyaline membranes	24	86%	2.43	0	0%	0.00	****	***<0.0001***	****	***<0.0001***
Alveolar fibroblast proliferation	14	50%	1.45	2	15%	0.69		*0.21*	*	***0.03***

^ Severity was scored from H&E stained tissue sections on a scale from 0 (trace) to 4 (most severe).

## Data Availability

Data are provided within the article and supplement material. Additional data can be requested from the corresponding author.
